# Post-operative lactate levels predict rate of complications and length of stay following on-pump cardiac surgery

**DOI:** 10.1186/2197-425X-3-S1-A105

**Published:** 2015-10-01

**Authors:** I Norkiene, E Navickaite, T Jovaisa

**Affiliations:** Vilnius University, Clinic of Anaesthesiology, Vilnius, Lithuania; Vilnius University, Faculty of Medicine, Vilnius, Lithuania; Lithuanian University of Health Sciences, Clinic of Anaesthesiology, Kaunas, Lithuania

## Introduction

Hyperlactatemia (plasma lactate > 2 mmol/l or >18 mg/dl) can occur in the setting of inadequate tissue perfusion or hypoxia or have non hypoxic origin like impaired buffering systems or metabolic disorders. Numerous studies have proven lactate concentration to be a good marker of disease severity for ICU patients. A single centre study recently have demonstrated it's predictive value following cardiac surgery.

## Aims

The aim of our study was to establish the prevalence of hyperlactatemia following normothermic and mild-hypothermic cardiopulmonary bypass (CPB) and to determine whether normal lactate levels (NL), moderate hyperlactatemia (MHL) or severe hyper lactatemia (SHL) at ICU admission was associated with a different postoperative outcomes.

## Methods

Retrospective analysis of consecutive normothermic and mild-hypothermic CPB cases performed in Vilnius University Hospital Stanatriskiu Clinics during the 4-month period in 2014. Based on previous publication we defined groups according to post-operative lactate levels - NL group < 1.6 mmol/l, MHL group 1,6-4,4 mmol/l and SHL group ≥ 4.4 mmol/l. We analysed the impact of hyperlactatemia on the length of post-operative ICU and hospital stay and also on the incidence of infectious and non-infectious complications. STS derived 30-day morbidity endpoints were used: permanent stroke, renal dysfunction or renal failure requiring dialysis, any cardiac re-operation and lung ventilation for more than 48 hours.

Statistical analysis was performed using MS Office Excel and GraphPad Prism 6 software.

## Results

Data of 271 consecutive patients was analysed. Medium age of the cohort was 65 ± 10,36 years, 180 (66%) of patients were male, 91(33%) - female. Normal lactate levels postoperativelly were detected in 127 (46,86%) patients, moderate hyperlactatemia in - 105 (38,75%), and severe elevation of lactates in - 39 (14,39%) patients.

The overall incidence of postoperative infectious complications was 12(9%) in NL group, 20(19%) in MHL and 12(31%) in SHL accordingly (p = 0,0003). Sternal would infection was significantly more frequent in MHL group (7(6%) vs. 9(9%) vs. 0(0%), p < 0,0001). The distribution of non infectious complications amongst three groups was 47(37%), 41(39%) and 24(62%) p = 0,0391. There was no significant difference between groups regarding development of single postoperative non-infectious complications. The longest post-operative ICU stay of 7 ± 8,62 days, was observed in SHL group (p < 0,0001). Overall post-operative in hospital stay was 14 ± 15,47 days in NL group, 16 ± 13,84 days in MHL and 18 ± 13,01 days in SHL group (p = 0,0002). There was a significant difference in mortality rates between groups 1(1%) v 1(1%) v 2(5%), p = 0,0018.

## Conclusions

Post-operative lactate level higher than 4,4 mmol/l was a significant predictor of worse post-operative outcomes (longer ICU and in-hospital stay, greater incidence of postoperative complications and increased mortality rates.

